# Editorial: Biomarkers and immunotherapy for genitourinary tumors

**DOI:** 10.3389/fimmu.2024.1394170

**Published:** 2024-03-22

**Authors:** Zeyu Han, Jianzhong Ai

**Affiliations:** Institute of Urology, Department of Urology, West China Hospital, Sichuan University, Chengdu, China

**Keywords:** genitourinary tumors, biomarkers, immunotherapy, bladder cancer, renal cell carcinoma, prostate cancer

The normal function of the genitourinary system is crucial for maintaining overall health, playing a vital role in metabolism and reproduction. However, due to rapid population growth and an aging trend, the incidence of three major genitourinary cancers including prostate cancer, bladder cancer, and renal cell carcinoma (RCC), has substantially increased, significantly impacting the global disease burden (1). Alongside surgical interventions, systemic therapies like chemotherapy, radiotherapy, and hormone therapy are employed in treating genitourinary tumors. Nevertheless, these approaches face limitations stemming from tumor heterogeneity, clinical variability, and drug resistance, often yielding unsatisfactory outcomes. In recent years, the emergence of immunotherapy, including immune checkpoint inhibitors (ICIs) and messenger RNA vaccines, has propelled precision medicine forward (2, 3). To date, the US Food and Drug Administration has approved ICIs for clinical use in certain genitourinary tumor patients (4). Biomarkers play a pivotal role in early tumor diagnosis, drug development, disease monitoring, and prognosis evaluation. However, current research on urogenital tumor biomarkers remains insufficient, hindering a comprehensive understanding of immunotherapy mechanisms. Therefore, delving into potential urogenital tumor biomarkers will aid in identifying therapeutic targets, enhancing the efficacy of immune and targeted therapies, and bolstering comprehensive patient management for urogenital tumors.

In recent years, the regulatory role of various cell death modes in tumors has garnered significant attention from researchers. Disulfide apoptosis, a novel form of regulated cell death induced by disulfide stress, has emerged as a focus of study (5). Recent research indicated that glucose deficiency triggers the accumulation of disulfide bonds in cytoskeleton proteins, thereby promoting cell death in SLC7A11-overexpressing cancer cells. Chen et al. employed consensus clustering to identify gene clusters and developed a prognostic model for disulfide-related genes. Validation using external datasets and experimental studies revealed that POU5F1 transactivates CTSE, thus promoting the development of bladder cancer cells. Anoikis, characterized by the detachment of cells from surrounding cells or stroma, results in cell death primarily through the regulation of apoptosis-related pathways (6). Similarly, Dong et al. identified nine anoikis-related genes using COX regression and other methods through analysis of the Cancer Genome Atlas (TCGA) and the Gene Expression Omnibus (GEO) databases, validating them *in vitro* via Reverse Transcription Quantitative Polymerase Chain Reaction (RT-qPCR). Zhang et al. designed a risk model based on long non-coding RNAs associated with anoikis to predict the prognosis of bladder cancer patients. Furthermore, Yuan et al. developed a copper poisoning-associated 11-gene signature model, demonstrating its efficacy as a predictor of survival time and response to Bacillus Calmette-Guérin (BCG)/ICIs in patients with bladder cancer. Wang et al. collected bladder cancer data from multiple databases to establish a prognostic signature for copper-related genes, which was subsequently validated in the GEO cohort. Their findings suggest that monooxygenase DBH-like 1 may play a role in the immune microenvironment of bladder cancer. Zhang et al. employed unsupervised clustering to identify molecules regulated by RNA modification writers in bladder cancer. They validated the established score using databases and clinical samples from hospitals. Population-based clinical studies have also provided valuable insights into the treatment and prognosis of bladder cancer. In a retrospective study by Xu et al., involving 25 patients with muscle-invasive bladder cancer who did not undergo radical cystectomy, the use of programmed cell death-1 (PD-1) inhibitors combined with radiotherapy or chemoradiotherapy yielded promising results. Ding et al. combined data from patients with non-muscle invasive bladder cancer from two institutions to develop a novel nomogram for individually assessing patient survival risk. Zhang et al. retrospectively collected data from 725 bladder cancer patients undergoing radical cystectomy and found that the preoperative systemic immune inflammation index can serve as a predictor of patient prognosis. Additionally, Liu et al. discovered that preoperative sarcopenia is predictive of response to intravesical BCG vaccination in non-muscle invasive bladder cancer patients. Establishing a risk-scoring model is crucial for improving the treatment and prognosis of bladder cancer. Insights into the cellular and molecular mechanisms can deepen our understanding of the biological processes underlying bladder cancer. Furthermore, independent prognostic factors identified from population-based clinical studies can help inform clinical decision-making.

The Research Topic comprehensively covers the pathogenesis, pathological classification, diagnosis, treatment, and prognosis of RCC. In the tumor immune microenvironment, Zhang et al. conducted cell experiments revealing elevated expression of tumor-associated M2 macrophages in RCC tissues. These macrophages were found to promote RCC development by regulating the expression of related genes. Intestinal flora’s role in tumor occurrence, particularly its relationship with urinary tract tumors, has garnered attention from researchers. Yang et al. conducted a review of previous studies and summarized the latest evidence on the mechanisms of intestinal and urinary microbiota in renal cancer development and treatment. They proposed that the abundance of microbiota correlates with drug efficacy and that certain microbiota may serve as markers of immune efficacy in renal cancer treatment. In pathological classification, Wu et al. utilized logistic regression analysis of preoperative indicators from 280 RCC patients. Their findings suggest that the preoperative neutrophil-to-lymphocyte ratio can distinguish sarcomatoid RCC from clear cell RCC (ccRCC). Xu et al. also discovered an association between the neutrophil-to-lymphocyte ratio and the prognosis of cancer patients. In terms of diagnosis, Li et al. conducted RT-qPCR on 224 subjects to detect microRNA (miRNA) expression levels. Combined with bioinformatics, they proposed that a combination of four miRNAs in serum could serve as a non-invasive diagnostic biomarker for RCC. Treatment modalities for RCC encompass various approaches, including cell death induction, antibiotics, and modulation of the coagulation system. In addition to the previously mentioned anoikis, immunogenic cell death (ICD) denotes tumor cell demise induced by certain drugs, radiation, and other interventions (7). Dead cells release signaling molecules, activating anti-tumor immunity. Wang et al. and Hao et al. delved into ICD and anoikis, respectively, offering fresh perspectives on the treatment and prognosis of ccRCC. Zheng et al. analyzed the differentially expressed genes of ccRCC, COVID-19, and berberine, revealing the relationship between potential targets and ccRCC and COVID-19 through a comprehensive series of analyses. Yin et al., employing single-cell sequencing and other methodologies, established a signature of coagulation-related genes in ccRCC, offering a potential avenue for clinical treatment. Xiong et al. investigated the distinctive role of the basement membrane gene in ccRCC and observed that low FREM2 expression correlates with a poor prognosis. Su et al. conducted a comprehensive review examining the relationship between the efficacy of ICIs and various components of the tumor microenvironment in renal cancer. Their findings serve as a valuable reference for the advancement of renal cancer immunotherapy. In evidence-based medicine, a meta-analysis conducted by Li et al., pooling data from 8 studies, concluded that cytoreductive nephrectomy is associated with better overall survival compared to the no cytoreductive nephrectomy group.

The occurrence and progression of prostate cancer are influenced by various factors, with gene mutation and aging recognized as significant contributors. Liu et al. conducted next-generation sequencing on 200 prostate cancer tissues and 714 blood samples, identifying common somatic abnormalities such as TP53, PTEN, and KRAS, along with common germline abnormalities including BRCA2, NBN, and ATM. They also observed a correlation between Gleason score and somatic aberrations of TP53, as well as positive germline aberrations of BRCA2/ATM. Han et al. extracted aging-related genes from TCGA and CellAge databases to construct an aging-related prognostic model for prostate cancer patients. The risk score from this model positively correlates with tumor mutation burden and immune checkpoints, aiding in treatment strategy selection for prostate cancer patients. It’s noteworthy that other genitourinary tumors have also been addressed in this context. Penile cancer and germ cell tumors are rare, with traditional therapies limited by tumor metastasis and toxic side effects. Immunotherapy offers promise for more precise treatment approaches (8). Tang et al. indicated the high expression of programmed death-ligand 1 in penile cancer tissue, supporting the use of specific immunotherapy. Additionally, therapeutic human papillomavirus vaccines and adoptive T-cell therapy hold potential as immunotherapy modalities for penile cancer patients. Schepisi et al. highlighted the challenges in applying ICIs to testicular germ cell tumors while pointing out that Chimeric Antigen Receptor T-cell therapy presents a new avenue for immunotherapy. Telomerase activation is implicated in the onset and progression of urothelial carcinoma, with telomerase reverse transcriptase-associated gene alterations expected to find clinical utility in managing urethral cancer. Liu et al. reviewed the variances in senescence among different epithelial cell types and explored the impact of telomerase reverse transcriptase regulatory mechanisms on urothelial carcinoma immunotherapy. Tang et al. conducted targeted next-generation sequencing on patients with upper tract urothelial carcinoma, evaluating the mutational profile using the COSMIC database to unveil the role of genomic mutational landscapes in urothelial carcinogenesis. Zheng et al. reported a case of metastatic primary urethral carcinoma with low expression of human epidermal growth factor receptor 2, in which the patient achieved sustained partial remission following treatment with PD-1 inhibitors and antibody-drug conjugates.

Innate and adaptive immune responses are pivotal in anti-tumor defenses. The discovery of biomarkers in genitourinary tumors holds profound significance in advancing precise and individualized immune treatments. In conclusion, this Research Topic contributes to elucidating the mechanisms and applications of biomarkers and immunotherapy in genitourinary cancers.

## Author contributions

JA: Writing – review & editing, Writing – original draft, Supervision, Resources, Methodology, Funding acquisition, Conceptualization. ZH: Writing – original draft, Validation, Investigation.
